# The Structure of OMCI, a Novel Lipocalin Inhibitor of the Complement System

**DOI:** 10.1016/j.jmb.2007.03.064

**Published:** 2007-06-08

**Authors:** Pietro Roversi, Olga Lissina, Steven Johnson, Nurfilza Ahmat, Guido C. Paesen, Kerstin Ploss, Wilhelm Boland, Miles A. Nunn, Susan M. Lea

**Affiliations:** 1 Sir William Dunn School of Pathology, University of Oxford, Oxford OX1 3RE, England, UK; 2 NERC Centre for Environmental Hydrology, Mansfield Road, Oxford OX1 3SR, England, UK; 3 Max Planck Institute for Chemical Ecology, Department of Bioorganic Chemistry, Hans-Knöll-Straße 8, D-07745 Jena, Germany

**Keywords:** OmCI, tick, complement C5, complement inhibitor, lipocalin

## Abstract

The complement (C) system is a potent innate immune defence system against parasites. We have recently characterised and expressed OmCI, a 16 kDa protein derived from the soft tick *Ornithodoros moubata* that specifically binds C5, thereby preventing C activation. The structure of recombinant OmCI determined at 1.9 Å resolution confirms a lipocalin fold and reveals that the protein binds a fatty acid derivative that we have identified by mass spectrometry as ricinoleic acid. We propose that OmCI could sequester one of the fatty acid-derived inflammatory modulators from the host plasma, thereby interfering with the host inflammatory response to the tick bite. Mapping of sequence differences between OmCI and other tick lipocalins with different functions, combined with biochemical investigations of OmCI activity, supports the hypothesis that OmCI acts by preventing interaction with the C5 convertase, rather than by blocking the C5a cleavage site.

## Introduction

Complement (C) is a potent, frontline, innate immune defence system that comprises approximately 30 fluid-phase and membrane-associated proteins.^1^ C activation results in the release of the acute inflammatory peptide mediators C3a and C5a^2^ by the C3 and C5 convertases, and formation of the terminal membrane attack complex (MAC, comprising C5b–C9) which can lyse cells directly, or trigger a variety of cellular activation events.^3^ Activated complement can inflict damage to the body's own tissues and has been implicated in numerous inflammatory disorders.^4^ Activation occurs *via* a tightly regulated proteolytic cascade, which is dependent on conformational changes induced by multi-protein complexes and by the cleavage events themselves. Additional regulation is achieved by both the short half-lives of activated C components and (in humans) more than 14 serum and cell-surface C regulatory proteins. Although the functional roles of C proteins are broadly understood, relatively few C component structures have been described, and even fewer atomic interactions elucidated in detail.^5,6^

Parasites that fail to control C activation may be damaged or killed by the host's inflammatory response, and by elaboration of the immune response orchestrated by complement. Most parasites express specific inhibitory proteins, or produce physical barriers and/or sequester host regulatory molecules to counteract C activity.^7–10^ The ticks, obligate ectoparasites (Acari, Parasitiformes), counteract harmful effects of C by secreting inhibitors into their feeding site.^11,12^ We have recently characterised OmCI, a 16 kDa protein derived from the soft-tick *Ornithodoros moubata* that specifically binds C component C5 in solution, prevents cleavage of C5a from C5, and thus inhibits formation of the MAC.^13^ OmCI belongs to a family of about 20 tick lipocalins that sequester mediators of inflammation from the host plasma.^14^ On the basis of sequence homology, a subfamily of tick lipocalins comprising the tick salivary gland proteins 1–3 (TSGP1–TSGP3) from the soft tick *Ornithodoros savignyi*, and moubatin from *O. moubata*, are likely to share the same molecular architecture as OmCI,^15^ but no structure is available for any member of this subfamily.

OmCI is the first parasite molecule described that specifically targets C5, a member of the C3/C4/C5 protein family that orchestrate the assembly of the terminal C multiprotein complexes. Only one other natural C5 inhibitor is known to date, the *Staphylococcus aureus* protein SSL7.^16^ Mature C5 comprises an α and β chain (115 kDa and 75 kDa, respectively) associated *via* a disulphide bond. Figure 4(c) illustrates the two-chain structure of the molecule. In a step crucial to terminal complement pathway activation, C5 is cleaved by the trimeric alternative and classical pathway C5 convertases (C3bBbC3b and C4bC2bC3b, respectively) at the peptide bond between residues R751 and L752. This cleavage splits off the N-terminal domain of the C5α chain, which is called the C5 anaphylotoxin or C5a (orange in Figure 4(c)) from the rest of the molecule, a much larger fragment called C5b. Following cleavage, C5b transiently gains the ability to interact with C6, and the C5bC6 complex is the hub for sequential assembly of C7, C8 and C9 that form the MAC.

Structures for the C3a, C3d and C4d fragments^17–19^ have been known for some time; the structures of whole C3^20^ and its fragments C3c^20^ and C3b^21–23^ were determined more recently. Details of the interactions between C3/C4/C5 family members remain undefined, and only a few small, independently folding domains within C5 have been described. These include NMR solution structures for the C5a fragment (8 kDa),^24,25^ and for the 17 kDa C-terminal C345C domain of the α chain of C5b (cyan in Figure 4(c)).^26^ The C345C domain of C5 appears to be multifunctional, with roles in both C5 cleavage and MAC assembly.^27,28^ It interacts with the C5 convertase;^26^ the C5 C345C residues that are critical for the interaction with the C5 convertase cluster on a mobile hydrophobic loop that protrudes from the domain.^26,29^

C5 inhibition with a humanized anti-C5 antibody forms the basis of a successful therapy against nocturnal paroxysmal hemoglobulinuria.^30,31^. Anti-C5 antibodies are also being developed to fight complement-mediated tissue damage in a range of clinical contexts, such as myocardial infarction,^32^ cardiac surgery,^33^ and systemic lupus erythematosus.^34^

OmCI prevents cleavage of C5 by both the classical and alternative C5 convertases^13^; its half life *in vivo* in mice is about 30 h, due to its high-affinity binding to C5 with a slow dissociation rate;^35^ no detailed knowledge is available about the exact site(s) of interaction between C5 and its convertases but, since OmCI inhibits human, mouse^35^ and guinea pig C, the inhibitor must bind to an element or elements of C5 that are conserved between species. We present here the atomic structure of OmCI, determined by X-ray diffraction at 1.9 Å resolution; the OmCI construct studied here is the double mutant N78Q/N102Q that allowed expression in *Pichia methanolica* (see Materials and Methods) and is termed pOmCI. We used biochemical and structure-based analyses to formulate hypotheses about the molecular basis of OmCI activity. This work is the first step toward the elucidation of OmCI C inhibition, and contributes to current investigations of the therapeutic potential of the inhibitor.

## Results

### Atomic structure for OmCI

**Figure 1:**
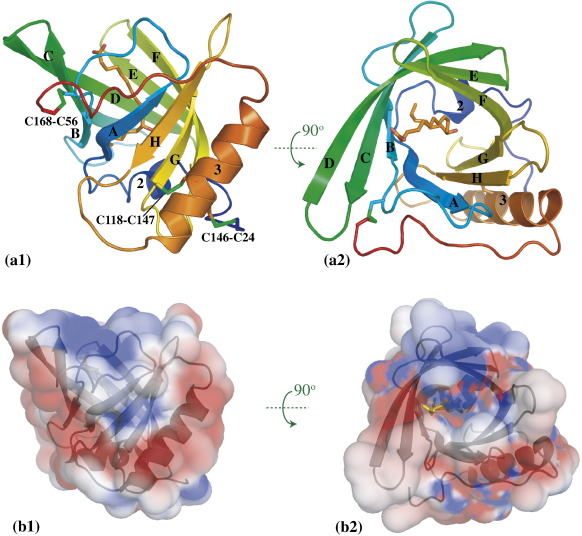
(a) Cartoon representations of the OmCI molecule from the *P*2_1_2_1_2_1_-A crystal form. Colour: blue to red, from N terminus to C terminus. The cysteine side-chains and the ricinoleic acid ligand are shown in stick representation, with C atoms coloured orange, O red and S green. The strands and helices are labelled following the tick lipocalin nomenclature.^36^ (b) OmCI molecular surface coloured by electrostatic potential, in the same orientations as in (a). Contours: − 2 kT/e, red; + 2 kT/e, blue. The pictures were produced with the PyMol [http://www.pymol.sourceforge.net/]. The electrostatic potential was computed with the program APBS^42^, run within PyMol, with the following parameters: ε_protein_ = 2.0; ε_solvent_ = 78.0; ionic strength, 150 mM NaCl. In (a) and (b), views 1 and 2 are rotated by 90° around the horizontal axis.

**Figure 2:**
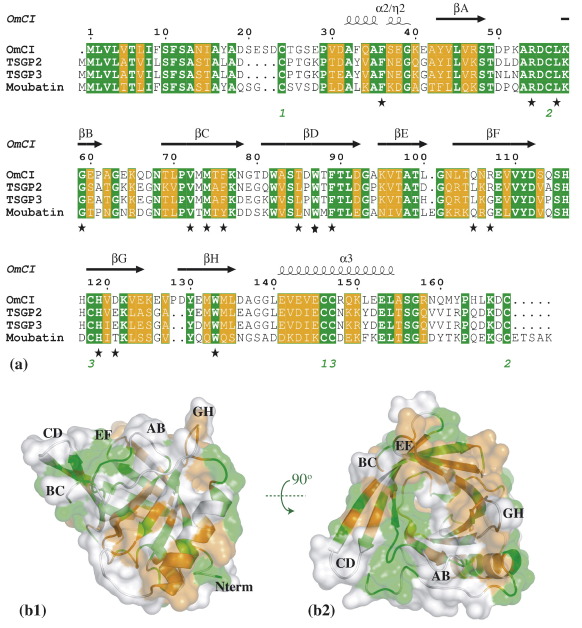
(a) The annotated sequences of OmCI (Uniprot Entry Q5YD59) and its closest soft tick protein homologues, TSGP2 and TSGP3 from *O. savignyi* (UniProt Entries Q8I9U1 and Q8I9U0) and moubatin from *O. moubata* (UniProt Entry Q04669). White font on green background, identical residues; white font on orange background, Risler similarity score ^55^ greater than 0.7. The secondary structure of OmCI is displayed above the sequences: beta sheets are symbolised by arrows, and helices by wiggly lines; below each cysteine residue, the disulphide bridge the cysteine residue belongs to is labelled in green (disulphide bridge1, Cys24–Cys146; disulphide bridge 2, Cys56–Cys168; disulphide bridge 3, Cys118–Cys147); starred residues are involved in contact with the ligand. The N-terminal residue of mature OmCI is D19. The Figure was prepared with the computer program ESPript.^56^ (b) The cartoon and surface representation of the OmCI residues that show conservation (green) or similarity (orange) in the family of proteins whose sequences are given in (a). Protein orientations are the same as in Figure 1. For reference purposes, the N terminus and a few of the loops are labelled.

OmCI displays a standard lipocalin fold (Figure 1), with a central eight-stranded antiparallel β barrel, flanked by the C-terminal helix (labelled 3 in Figure 1). As seen in hard-tick lipocalins,^14,36^ this helix is pinned to the barrel by a disulphide bond (C118–C147, Figure 1(a)), while a second disulphide bond links the terminal cysteine residue to the loop between the first two strands (C56–C168, Figure 1(a)). The N-terminal portion, containing a mixed α/3_10_ helix (labelled ‘2’ in Figure 1 and αA/ηA in Figure 2(a)), closes the bottom of the β-barrel; the αA/ηA helix is interrupted in the middle at residue F36, which is involved in ligand binding (see below). The main novel structural feature of OmCI is the extra disulphide bond C24–C146 (SS bond number 1 in Figure 2(a)), which ties the N-terminal portion to helix α3; this disulphide bond was predicted by Mans *et al.* for OmCI sequence homologues.^15^ Thus, the mature protein (starting at residue 18) is tethered by disulphide bonds close to both the N and the C terminus, a feature that may confer extra resistance to proteases. No ordered electron density is visible in the crystal for the first four residues at the N terminus (sequence DSES): the traceable electron density starts at D23, the residue immediately preceding the first cysteine.

### The pocket and the lipid

**Figure 3:**
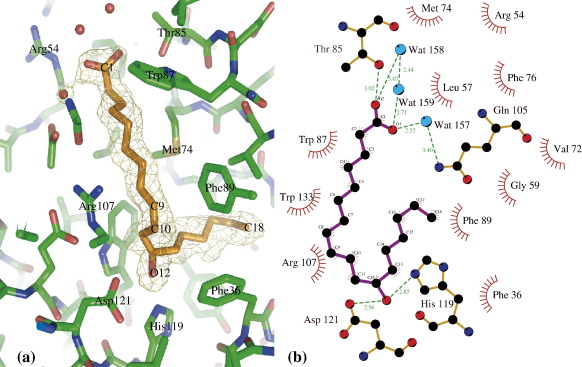
(a) P2_1_2_1_2_1_-A 1.9 Å *F*_o_–*F*_c_ electron density, contoured at the + 3.0 σ level, computed before any modelling of the ligand was attempted. The final model for the ricinoleic acid ligand is shown with C (orange) and O(red) surrounded by the OmCI pocket residues (C, green; O, red; N, blue; and S, yellow); the picture was produced with PyMol [http://www.pymol.sourceforge.net/]. (b) A representation of the water molecules and OmCI residues forming hydrogen bonds (with distances in Å) and non-bonding hydrophobic contacts to the ricinoleic acid. The picture was produced with the program LigPLot.^57^

The pocket at the centre of the pOmCI β-barrel in the crystal contains a ligand, a model for which was initially built in the residual electron density as a C_18_ fatty acid (see Figure 3(a) for the electron density used for the first model building). GC-MS after extraction from a pOmCI sample and esterification (see Materials and Methods) identified the methyl ester of ricinoleic acid *(*(R)-12-hydroxy-*cis*-9-octadecenoic acid), C_18_H_34_O_3_, as the major product (47%); minor components were identified as tetraethyleneglycol diethylether (21%), palmitic acid methyl ester (21%), and stearic acid methyl ester (11%). The presence of palmitic acid and stearic acid methyl esters was confirmed by GC-MS analysis of pure samples of the same esters. Tetraethyleneglycol diethylether is commonly observed when plastics have been used to store samples. The methyl ester of ricinoleic acid displayed characteristic even-numbered fragment ions at *m*/z 294 (*M*^+^-H_2_O), *m*/*z* 198, *m*/*z* 166, *m*/*z* 124, and *m*/*z* 74, facilitating identification. Moreover, the methyl ester of the protein-associated fatty acid and the methyl ester of authentic ricinoleic acid eluted at identical retention times and showed coincident mass spectra.

The most likely origin of the ricinoleic acid bound to pOmCI in the crystals is the medium used for expression of the recombinant protein,^13^ as a sample of native OmCI purified from tick salivary gland extract did not show any ricinoleic acid, nor significant amounts of other ligands when analysed by GC-MS (data not shown).

In the pOmCI crystals, the carboxylic moiety of ricinoleic acid faces the solvent at the opening of the β-barrel, where it forms a hydrogen bond with T85, and interacts with the positively charged head of R54. The rest of the molecule fits snugly into the L-shaped pocket, with a kink at position 12, where the hydroxyl group is hydrogen bonded to H119 and D121 (see Figure 3(b)). The C_9_ = C_10_ double bond stacks against the guanidinium head of R107. These interactions, together with other hydrophobic protein–ligand contacts with the side-chains of residues F36, L57, G59, V72, M74, F76, W87, F89 and W133, account for a contact area of about 280 Å^2^ (as computed with the program CCP4-areaimol^37^).

### Structural and sequence homologues

A DALI search^38^ using the OmCI coordinates on the Protein Data Bank as of April 2006 returns the tick histamine-binding protein HBP (PDB ID 1QFT, rmsd 1.2 Å over 103 C^α^ atoms ; this is the model that was used to determine the OmCI structure by molecular replacement) and the *Escherichia coli* putative phospholipid-binding lipocalin BLC (PDB ID 1QWD, rmsd 1.4 Å over 77 C^α^ atoms), plus a number of other lipocalins, as the closest OmCI structural homologues. However, sequence similarity and especially conservation of the cysteine residues, puts proteins of unknown structure closer to OmCI: the tick salivary gland proteins 1, 2 and 3 (TSGP 1–3) from the soft tick *Ornithodoros savignyi*,^39^ with sequence identities in the range 38.5–58%; and moubatin from the soft tick *O. moubata* (sequence identity 49%).^40^ Most of the sequence differences between OmCI and these sequence homologues localise to loops between the central strands of the barrel (see Figure 2(b)). More distant relatives of OmCI that still preserve all the cysteine residues are *O. savignyi* TSGP4 (22% sequence identity) and Arg-r-1, an allergen from *Argas reflexus* (24% sequence identity).^41^ (An up-to-date list of all the family members is available as Supplementary Data).

### Analysis of the surface properties of OmCI structure

The electrostatic potential around the OmCI molecule was evaluated with the program APBS,^42^ and is shown in Figure 1(b). The most significant features are a positively charged patch at the brim of the fatty-acid-binding pocket, which is mainly due to residue K65; and negative patches on the opposite face of the molecule, localised on the 3_10_ helix turn and its surroundings, and on the side of the barrel, on helix α3.

### Structural similarity to C8γ and C5-C345C domain

While the closest OmCI structural homologues are tick proteins, the protein also bears intriguing structural similarity to two complement protein domains, namely C8γ and the C345C domain of complement proteins C3, C4 and C5.

C8γ is a 22 kDa domain of complement component C8. Although C8γ is not essential for complement-mediated lysis, C8 lacking this domain shows impaired activity.^43^ C8γ has a lipocalin fold,^44^ and its overall size and shape, as well as its hydrophobic pocket, are very similar to those of OmCI (2.8 Å rmsd over 91 C^α^ atoms). Although physiological ligands have yet to be identified, both OmCI and C8γ are likely to bind fatty acids or related compounds. As was hypothesised for C8γ,^44^ OmCI may bind inflammatory response mediators or modulators; these molecules are likely to be produced at the site of complement activation during tick feeding, and by sequestering them *via* OmCI, the tick would interfere with the host inflammatory response.

The other complement protein domain that OmCI shows structural similarity to is the C345C domain of complement components C3, C4 and C5. This domain is involved in interactions between terminal components of complement; in particular, it has been shown to be essential for C5 cleavage by the C5 convertases and for C5 activation.^29^ When superimposing OmCI onto the NMR structure of the C345C domain of C5, PDB ID *1XWE*, five β strands of OmCI (BCDAH) align with five β strands of C5 C345C (ABCDE^2^),^26^ with rmsd of 2.0 Å over 44 C^α^ atoms. In this superposition, the DE loop of the C5 C345C domain that is necessary for C5 cleavage and activation is overlaid by the OmCI EF loop, one of the sites of sequence difference between OmCI and its sequence homologues (see Figure 2).^29^

### A model for the interaction of OmCI with C5

On the basis of the C5 C345C and OmCI structural similarity, we propose a mechanism for the C5:OmCI complex that involves OmCI displacing the C5 C345C domain from the surface of C5. For this model to be correct, the linker between the C5 C345C domain and the rest of the C5 molecule needs to be flexible enough to allow the displacement of the C345C domain by the OmCI molecule. This is supported by a number of observations based on the structures of the C5 homologues C3 and C3b: (i) the contact area of the C3 C345C domain with the rest of the molecule in the C3 structure is only 960 Å^2^, with an estimated energy for the interaction of less than 5 kcal/mol;^45^ (ii) the relative orientation of the C3 C345C domain with respect to the main body of the molecule shows a large degree of flexibility when comparing the structure of C3 with those of C3b (PDB ID 2I07) and C3b:CRIg complex (PDB ID 2ICF): in the C3b structure the C3 C345C domain is swung out by 22° and moves by about 20 Å away from the position it occupies in C3; in the C3b:CRIg complex, the rotation is about 35° and the shift about 37 Å away from the C3 position; (iii) the anchor region (residues 1475–1495) securing the C3 C345C domain to the main body of C3 undergoes large conformational changes upon going from C3 to C3b.^21–23^

**Figure 4:**
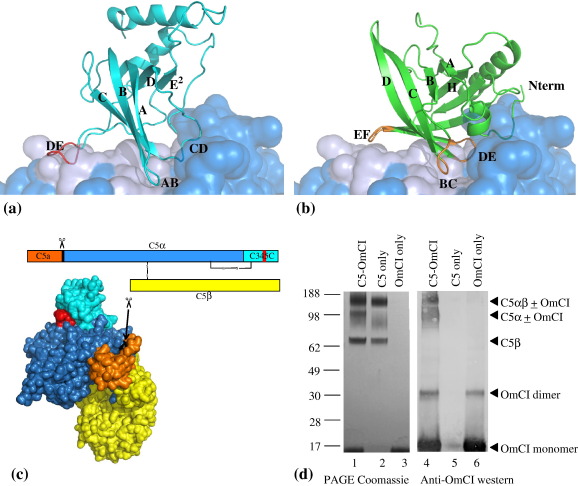
(a) A model for the region of C5 around its C-terminal C345C domain. In cartoon representation, the NMR structure of the C5 C345C domain (coloured cyan, from PDB ID 1XWE). In surface representation, coloured blue, a homology model for the neighbouring surface of the C5 molecule (excluding the C345C domain) based on the structure of C3, PDB ID 2A73. The contact areas of this homology model for the rest of C5 with the C5 C345C domain are coloured light blue. The C5 C345C DE loop critical for the interaction with the C5-convertase (C5 residues 1622–1640) is coloured red. (b) The proposed model for the complex between OmCI and C5. In cartoon representation, the structure of OmCI (green), superposed on the NMR model of the C5 C345C placed as described above (see (a)). The homology model for the C5 surface (without the C345C domain) is coloured as in (a). The OmCI loops BC, DE and EF are coloured orange. (c) A representation of the two chain structure of C5, modelled after the C3 crystal structure, PDB ID 2A73. The disulphide bond linking the C5α and C5 α chains, and the one linking the C345C domain to the main body of the C5 α chain, are symbolised by black lines. The N-terminal domain of the C5 α chain (the C5 anaphylotoxin, (C5a)) is coloured orange; the C-terminal domain of the C5α chain (the C345C domain) is coloured cyan; the DE loop of the C345C domain (see (a)) is coloured red; the rest of the C5 α chain is coloured blue. The C5 β chain is coloured yellow. Residues R751–L752 where the cleavage of the C5α chain occurs are coloured black and are indicated by the arrow. (d) SDS-PAGE gels of the BS3 crosslinking mixtures (see Materials and Methods). Left-hand side lanes: gel stained with Coomassie brilliant blue. Right-hand side lanes: Immunoblot with anti-OmCI antibodies. Lanes 1 and 4, crosslinking in the presence of both OmCI and C5; lanes 2 and 5, crosslinking in the presence of C5 only; lanes 3 and 6, crosslinking in the presence of OmCI only. The upward shift in C5α, but not C5α, in the presence of OmCI (lanes 1 and 4), indicates crosslinking of OmCI to C5α. (a), (b) and (c) were produced with the program PyMol [http://www.pymol.sourceforge.net/].

To further refine our hypothesis, we first generated a homology model for the C5 structure, based on the structure of C3 (28.2% identity and 48.3% similarity); and then incorporated the NMR structure of the C5 C345C domain in this model by substituting it for the same domain of the C5 homology model (see Figure 4(a)). In this C5 model, the DE loop of the C345C domain that is necessary for C5 cleavage and activation is one of the three contact points orienting this domain with respect to the rest of C5.^29^ The contact area of the C5 C345C domain with the rest of the C5 molecule in this model for C5 is 880 Å^2^.

Once this model for C5 was obtained, we built a model for the interaction of OmCI with the main body of C5 by overlaying the OmCI crystal structure on the C5 C345C domain placed as above, and displacing the C5 C345C domain (see Figure 4(b)). Both of the main sequence insertions by which OmCI differs from its closest homologues, located at the N terminus and at loop GH (see Figure 2), are in proximity of the main body of C5 in this orientation. The other two OmCI loops that are in close contact with the surface of C5 in this model are OmCI loops BC and DE. The contact area of OmCI with the rest of the C5 molecule in this model is 1080 Å^2^, slightly larger than that computed for the C5 C345C domain and the main body of C5.

In summary, we hypothesise that (i) the C345C domain of C5 is in similar placement with respect to the main body of the molecule to the C345C domain of C3; and that (ii) the structural similarity of OmCI to the C5 C345C domain is of functional significance. On the basis of these hypotheses, we propose that OmCI inhibits C5 cleavage by dislodging the C5 C345C domain from the position needed for the C5 convertase to approach and/or cleave. This model for the C5:OmCI interaction implies that several lysine residues in OmCI would lie close enough to lysine residues in C5α to allow crosslinking with bis(sulfosuccinimidyl)suberate (BS^3^) (cross-linking distance 12 Å). This prediction is consistent with the results of our BS^3^ crosslinking experiments, which show a mobility-shift of the C5α but not the C5β chain and, therefore, indicate binding of OmCI to C5α and not to C5β (see Figure 4(d)).

## Discussion

When OmCI isolated from the tick saliva was analysed by GS-MS, no detectable trace of small-molecule ligands bound to it are found: this suggests that the presence of a ligand in the pocket is not critical for folding nor stabilisation of the folded protein. On the other hand, ricinoleic acid interacts rather specifically with residues H119 and D121 (with the OH group) and R107 (with the C_9_ = C_10_ double bond). Given the good complementarity between ricinoleic acid and the central pocket, it is likely that any physiological ligand(s) of OmCI will be lipid or lipid derivatives bearing some similarity to ricinoleic acid (e.g. a negatively charged head group, an unsaturation at C_9_ = C_10_ and a hydrogen bond donor at position 12). Assuming OmCI acquires its natural ligand(s) from the host plasma during the tick's blood meal, derivatives of arachidonic acid such as leukotrienes, prostaglandins and thromboxanes are possible, physiologically relevant OmCI ligands. By sequestering such molecules, OmCI may further reduce inflammation at the feeding site. Until the exact identity of the OmCI ligand(s) *in vivo* and their role in the interaction between the tick parasite and the host are elucidated, OmCI remains an orphan lipocalin.

OmCI's tick lipocalin sequence homologues (see Figure 2), TSGP 2 and TSGP 3 have no putative function as yet, while moubatin is reported to be a platelet aggregation inhibitor. The most significant sequence differences in the residues lining the pocket between moubatin and OmCI are the substitution of Leu for Phe at residue 33 and Gly for Arg at residue 107, both of which render the surface of the moubatin pocket larger (by an estimated 100 Å^2^). On the basis of our OmCI structure, we propose that in moubatin the pocket may be large enough to bind prostanoids (prostaglandins and thromboxanes) involved in platelet aggregation.^46^

The structural similarity of OmCI to the C5 C345C domain leads to the hypothesis that OmCI inihibits C5 cleavage by dislodging the same domain from the surface of C5, thereby disturbing the approach of the C5 convertase. This model is consistent with the published evidence that the DE loop of the C5 C345C domain is necessary for C5 cleavage and activation,^29^ and with the results of our crosslinking experiments that demonstrate OmCI binds to the C5α chain. This model lends support to the hypothesis that OmCI acts by preventing interaction with the C5 convertase, rather than by blocking the C5a cleavage site.^13^ Further biochemical and structural characterisation of the C5:OmCI interaction are in progress.

## Materials and Methods

### Mutation of the glycosylation sites, cloning into pMETαC, an expression and purification from *Pichia methanolica*

Native, mature, OmCI is not glycosylated but its recombinant version expressed in *P. methanolica* is hyperglycosylated.^13^ The asparagine residues of the NX(S/T) glycosylation sites (N78 and N102) were therefore mutated into glutamine, and expressed in *P. methanolica* and purified as described.^35^ In the following, we refer to this double mutant N78Q/N102Q *Pichia*-expressed protein as pOmCI.

### Crystallization and X-ray diffraction

All crystals were grown by the vapour-diffusion, sitting-drop method at 20 °C; screens were set up using a crystallisation robot (TECAN, UK). The crystallisation drops were obtained by mixing 0.2 μl of protein solution with 0.2 μl of the crystallisation screen, and were equilibrated against 100 μl of mother liquor. pOmCI crystals grew by mixing the protein solution at 25.6 mg/ml with either MD Structure Screen I condition 2 (30% (w/v) PEG 4000, 0.1 M sodium acetate (pH 4.6), 0.2 M ammonium acetate) or MD Screen I condition 14 (30% PEG 8000, 0.1 M sodium cacodylate (pH 6.5), 0.2 M ammonium sulphate) (Molecular Dimensions Structure, USA). The crystals took over a month to grow to dimensions of approximately 200 μm × 80 μm × 50 μm.

Diffraction data from the pOmCI crystals were collected at 100 K on beamline ID14-4 at the ESRF (see Table 1 for data collection statistics). The crystals were cryoprotected with 15% (v/v) glycerol in mother liquor. The crystals belong to space group *P*2_1_2_1_2_1_, with two slightly different sets of cell dimensions (*P*2_1_2_1_2_1_-A and *P*2_1_2_1_2_1_-B). Crystals of either kind grew in the same conditions and could be harvested from the same drop. All X-ray data integration and scaling were done using the computer programs Mosflm^47^ and Scala.^48^

### Structure determination, model building and refinement

Initial structure factor phases were obtained for the pOmCI crystals by molecular replacement, with the female-specific tick histamine-binding protein (PDB ID *1QFT*, 21% sequence identity^14^) as a search model, against the *P*2_1_2_1_2_1_-A data; the computer program AMoRe^49^ was run *via* the molecular replacement web server CASPR.^50^ Iterative model building and refinement with programs xfit^51^ and Buster-TNT^52^ completed the structure. An initial model for the bound fatty acid was built in the residual electron density, and its exact chemical nature adjusted to that of ricinoleic acid once the mass-spectroscopy result was available. Once complete, the *P*2_1_2_1_2_1_-A pOmCI model was rebuilt and refined against the *P*2_1_2_1_2_1_-B data, after an initial round of rigid-body refinement, again using xfit and Buster-TNT. Table 2 contains the final crystallographic refinement data and statistics.

### Isolation and GC-MS analysis of ricinoleic acid from the recombinant pOmCI

Purified pOmCI in PBS (200 μl; 8.6 mg/ml) was extracted with CHCl_3_ (130 μl; 1:1, v/v) and evaporated to dryness at room temperature with a gentle stream of nitrogen. A few drops of 6 M diazomethane in ether were added to methylate the free acid. After 10 min, the solvent and excess diazomethane were removed by a stream of nitrogen and the sample was taken up in 10 μl of dichloromethane. The sample was used directly for gas chromatography-mass spectrometry (GC-MS) analysis. The reference ricinoleic acid was likewise methylated by diazomethane before GC-MS analysis.

Mass spectrometric analysis of ricinoleic acid was performed on a TraceMS (ThermoFinnigan, D-63329 Egelsbach, Germany) equipped with fused silica Alltech EC5 (D-82008 Unterhaching, Germany) capillary column (15 m × 0.25 mm; 0.25 μm film thickness) using He at 1.5 ml min^− 1^ as the carrier gas. Samples (1 μl) were injected in the split-less mode (1 min) and separated under programmed conditions starting at 60 °C (1 min) followed by heating at 10 deg.C min^− 1^ to 180 °C, and at 4 deg.C min^− 1^ to 280 °C and held there for 2 min. Full scan spectra were measured in electron impact (EI) mode at 70 eV, with a source temperature of 200 °C, transfer line at 280 °C, and an emission current of 250 μA. The instrument was operated between *m*/*z* 30 and *m*/*z* 570 at 2 scans s^− 1^.

### C5 and C5:OmCI modelling

A homology model for human C5 (1681 residues) was built with the program Modeller,^53^ using the crystal structure of human C3 (1653 residues, PDB ID 2A73) as a template. The overall level of identity between the sequences of the two molecules is 28.2%, the overall similarity is 48.3%. The NMR model of the C5 C345C domain (PDB ID 1XWE) was superimposed onto the C5 C345C domain of the C5 homology model using the program LSQMAN,^54^ the same program was used to overlay the OmCI crystal structure onto the C5 C345C domain. The contact areas between the C5 C345C domain and OmCI with the rest of C5 in these models were evaluated with the EBI PISA server.^45^

### Crosslinking

Human C5 was purchased from Calbiochem. The C5–OmCI complex was prepared by adding 2.5 μg of pOmCI to 5 μg (1 μg/μl) of C5 (approximately fivefold molar excess of pOmCI), both dissolved in PBS, final volume 9.5 μl. The reaction mixture was incubated at room temperature for 15 min to allow the complex to form, before adding the cross-linker bis(sulfosuccinimidyl)suberate (BS^3^; Pierce) to a final concentration of 0.8 mM. For C5 crosslinking, BS^3^ was added to 5 μg of C5 in 9.5 μl of PBS; for OmCI crosslinking, BS^3^ was added to 2.5 μg of OmCI. After incubation for30 min, reducing SDS sample buffer was added. The reduced samples were electrophoresed on Novex Tris-Tricine gradient 4%–14% (w/v) polyacrylamide gels and stained with Coomassie brilliant blue or transferred to nitrocellulose and immunoblotted with (1:500, v/v) anti-OmCI polyclonal rabbit serum (a kind gift from Professor Paul Morgan, Cardiff University College Hospital, Wales, UK).

## Figures and Tables

**Table 1:** OmCI X-ray data collection and processing

Data set	*P*2_1_2_1_2_1_-A	*P*2_1_2_1_2_1_-B
X-ray source	ESRF ID14-4	ESRF ID14-4
Detector	ADSC Scanner	ADSC Scanner
Space group (Z)	*P*2_1_2_1_2_1_ (4)	*P*2_1_2_1_2_1_ (4)
Unit cell parameters		
*a* (Å)	45.17	45.09
*b* (Å)	55.52	54.00
*c* (Å)	60.62	55.03
α (deg.)	90.0	90.0
β (deg.)	90.0	90.0
γ (deg.)	90.0	90.0
Wavelength (Å)	0.9757	0.9792
Resolution limits (Å)	40.9–1.9 (2.0–1.9)	38.5–2.3 (2.4–2.3)
Completeness (%)	99.6 (99.3)	99.7 (99.7)
Measured reflections	110,764	58,577
Unique reflections	12,509	6331
Multiplicity	8.9 (9.1)	9.2 (7.9)
*R*_merge_	0.095 (0.371)	0.124 (0.362)
*I*/σ(*I*)	6.5 (1.6)	4.8 (1.8)

Values for the highest resolution shell are given in parentheses.

**Table 2:** OmCI crystal structure refinements statistics

Crystal	*P*2_1_2_1_2_1_-A	*P*2_1_2_1_2_1_-B
Resolution range	40.9–1.9 (2.0–1.9)	20–2.3 (2.4–2.3)
*R*_all_ (%)	17.1 (17.5)	19.7 (21.2)
*R*_work_ (%)	16.9 (17.3)	19.5 (21.1)
*R*_free_ (%)	21.0 (22.9)	23.4 (22.7)
rmsd from ideal		
Bond lengths (Å)	0.014	0.003
Bond angles (deg.)	0.8	0.6
Residues modelled (range)	145 (24–168)	146 (23–168)
Water molecules modelled	160	44
Non-protein molecules	One acetate ion; one ricinoleic acid	One acetate ion; one ricinoleic acid
Average *B* values		
Protein (Å^2^)	19.9	19.8
Ligands (Å^2^)	29.5	30.0
Water molecules (Å^2^)	33.0	19.2
Ramachandran plot		
Residues in favoured regions (%)	98.6	97.2
Residues in forbidden regions (%)	0.0	0.0
PDB identifier	2CM4	2CM9

Values for the highest resolution shell are given in parentheses.
