# Supplementary material for: The Structure of OMCI, a Novel Lipocalin Inhibitor of the Complement System
Source: J Mol Biol. 2007 Jun 8;369(3-3):784–93. doi: 10.1016/j.jmb.2007.03.064 (PMC2724154; doi:10.1016/j.jmb.2007.03.064)
Supplement: Supplementary material [file mmc1.doc]

Supplementary material for the article ”THE STRUCTURE OF OMCI, A NOVEL LIPOCALIN INHIBITOR OF THE COMPLEMENT SYSTEM” by ***Pietro Roversi, Olga Lissina, Steven Johnson, Nurfilza Ahmat, Guido C. Paesen, Kerstin Ploss, Wilhelm Boland, Miles A. Nunn & Susan M. Lea***

ClustalW alignement of other tick protein sequences homologous to OmCI

tr|Q09JF6|Q09JF6_9ACAR --------------------------------------------------

tr|Q09JV2|Q09JV2_9ACAR -MFLLLVFASVHFTCGYAAGSSPVRYKPTSVRDFKSYLEGKEGLKQYQDG 49

tr|Q09JV9|Q09JV9_9ACAR -----MVSVAIAVACLFAAVS--TEAS--APCNFNGPFQAWRSVNGPGSG 41

tr|Q09JR9|Q09JR9_9ACAR -----MVSVTLAVACLFAAVS--AAASS-AQCDFGGPFQAYKSVNGPGNG 42

tr|Q09JE7|Q09JE7_9ACAR -----MVATVVFVACLSAVLS--AEVSPDVQCNFTGPFSALRSVNGRGSG 43

tr|Q09JX9|Q09JX9_9ACAR -----MALIILLAACLS----------VATAQQQCDTVSAWQSLRGPGTG 35

tr|Q5GQ85|Q5GQ85_9ACAR -----MALIILLVACLS----------VVSADDCSGKTDAWTSIKGPKTG 35

tr|Q09JS0|Q09JS0_9ACAR MDSMGVVLKVFLLACLCSSIAAARRTRTTNVSGCDRQNDALRALKARNSS 50

tr|Q8I9U1|Q8I9U1_9ACAR ----MMLVLATVILSFSASTA---LAD----CPTGKPTDAYVAFN-EGQG 38

tr|Q8I9U0|Q8I9U0_9ACAR ----MMLVLATVILSFSASTA---LAD----CPTGKPTEAYVAFN-EGKG 38

tr|Q5YD59|Q5YD59_ORNMO -----MLVLVTLIFSFSANIA---YADSESDCTGSEPVDAFQAFS-EGKE 41

sp|Q04669|MOUB_ORNMO ----MMLVLTTLIFSFSASIA---YAQSG--CSVSDPLDALKAFK-DGAG 40

tr|Q09JN5|Q09JN5_9ACAR --------------------------------------------------

tr|Q09JR1|Q09JR1_9ACAR --------------------------------------------------

tr|Q8I9U2|Q8I9U2_9ACAR --MQRLLLLLIALFSLSCAEAG--------PDGCVGSTEAKVAVFGEGGN 40

tr|Q8I9T9|Q8I9T9_9ACAR ---MDCKLVAIALFIFSLDFAHAANDVWNVLKGSDSKFLMVKRTYERGAN 47

tr|Q09JJ8|Q09JJ8_9ACAR --------------------------------------------------

tr|Q09JL4|Q09JL4_9ACAR --------------------------------------------------

tr|Q09JF6|Q09JF6_9ACAR ---------------------------DCEKHTAFTEMEFYNTTSEKW-- 21

tr|Q09JV2|Q09JV2_9ACAR WKFLTTGKEMYLYQRSFQEDPKYGNKCKCVKSKHLTVKEEAQTVAADLSF 99

tr|Q09JV9|Q09JV9_9ACAR G-------------------------YYMVKTTDPQTPDCPYVLVPRTRL 66

tr|Q09JR9|Q09JR9_9ACAR G-------------------------YYLRKTTKPGTPECAYVLVPQNTL 67

tr|Q09JE7|Q09JE7_9ACAR G-------------------------YYLINTTENNRPNCTYVRAPEVRL 68

tr|Q09JX9|Q09JX9_9ACAR G-------------------------YYLFKTTEGGKTDCTYVKGSNFND 60

tr|Q5GQ85|Q5GQ85_9ACAR G-------------------------YWLKQTTKTGENECTYVKGTDFKE 60

tr|Q09JS0|Q09JS0_9ACAR L-------------------------YVLQQTTSGAGESCTYVKAQGFDE 75

tr|Q8I9U1|Q8I9U1_9ACAR A-------------------------YILVKSTDLDARDCLKGSATGKKE 63

tr|Q8I9U0|Q8I9U0_9ACAR A-------------------------YILVRSTNLNARDCLKGEATGKKE 63

tr|Q5YD59|Q5YD59_ORNMO A-------------------------YVLVRSTDPKARDCLKGEPAGEKQ 66

sp|Q04669|MOUB_ORNMO T-------------------------FLLQKSTDPQARDCLKGTPNGNRD 65

tr|Q09JN5|Q09JN5_9ACAR ---------------------------FQFKLQSVNNTYIRNALVSDNE- 22

tr|Q09JR1|Q09JR1_9ACAR ------------------------------MHSKNEEEGFVVHKMEFWDT 20

tr|Q8I9U2|Q8I9U2_9ACAR AGSPTIG------------------YSYLVKTTYPDEHACVYILPPYGTA 72

tr|Q8I9T9|Q8I9T9_9ACAR K-------------------------CVYMKRTSMDESSHTLEVLMGYSK 72

tr|Q09JJ8|Q09JJ8_9ACAR -----------------------------CVECEENVESWIYVNSSADPE 21

tr|Q09JL4|Q09JL4_9ACAR ----------------------------RLGRVRQSENYTTNNIMRLYEK 22

tr|Q09JF6|Q09JF6_9ACAR -------VSYTVPAKVVTEGKYSKPNVIRAGNETIGRY--------ADSP 56

tr|Q09JV2|Q09JV2_9ACAR LHENLTSVHFTVYFSVNKTGKESR--VIYASYEPEKPG--------FPFP 139

tr|Q09JV9|Q09JV9_9ACAR TEGDAVEFTYGSLEDGE--LTRRTATVSGQGSNIVVTGG----DNPGTTT 110

tr|Q09JR9|Q09JR9_9ACAR SEGQSTSFTYGKLQNGQ--MIQLTATVTVNGDKIEVTGAG--QDLSGTTT 113

tr|Q09JE7|Q09JE7_9ACAR TEGSREKFTYGQLKKGQNVMRRHRGRVHVQGDMIIVTGK-----RAGISQ 113

tr|Q09JX9|Q09JX9_9ACAR AAQTAT-YTYGNLGSGNQ-LTQQTASASISGNAIVVG--------TDHSE 100

tr|Q5GQ85|Q5GQ85_9ACAR NTKTAT-YTYGYKDASGK-LTKTTGTATAKGSDIVVG--------SDTST 100

tr|Q09JS0|Q09JS0_9ACAR AQRTAL-YIYGNATDNGT-TVSSWTQRVRAENDSIVGNKTLTPNVTERWE 123

tr|Q8I9U1|Q8I9U1_9ACAR GNKVPVMMAFKNEGQWVS----LPWTFTLDGPKVTATD----GQRTLKRE 105

tr|Q8I9U0|Q8I9U0_9ACAR GNTLPVMMAFKDEGKWVS----LPWTFTLDGPKVTATH----GQRTLKGE 105

tr|Q5YD59|Q5YD59_ORNMO DNTLPVMMTFKNGTDWAS----TDWTFTLDGAKVTATL----GNLTQNRE 108

sp|Q04669|MOUB_ORNMO GNTLPVTMTYKDDSKWVS----LNWMFTLEGANIVATLE---GKRKQRGE 108

tr|Q09JN5|Q09JN5_9ACAR -------------------------TTVNEGSAADDEK--------PPLI 39

tr|Q09JR1|Q09JR1_9ACAR RASRMAKVETRLTTKKSKGYKISNLLVPQLGPSQGPGGP------EAVFP 64

tr|Q8I9U2|Q8I9U2_9ACAR DASGRYPYRMGYKDSNDQ-WVKLDGKIKTEGSKIIDNDP---EYGDTVTT 118

tr|Q8I9T9|Q8I9T9_9ACAR AGTTTDFVEPSKYTVTATSEGASTYNMMTVRRGPASHG--------VKFE 114

tr|Q09JJ8|Q09JJ8_9ACAR RATVRS----------------STRSTTANQIVLHSEGG------QQLIY 49

tr|Q09JL4|Q09JL4_9ACAR KDPTKVFNYTGLYSDGK-------------GCIIVRAEHWG-----GKCE 54

tr|Q09JF6|Q09JF6_9ACAR IMFS-DYKTCDVVRAPHT--GNES--DCELWVAENYVDNYPS-CCDFIYD 100

tr|Q09JV2|Q09JV2_9ACAR VAFA-DYESCAVVRVPHRDKGKNQ--ACELWVYKDHVKRVKS-CCFFIFD 185

tr|Q09JV9|Q09JV9_9ACAR LIFS-DYQTCDVVRGPTG--------GYELWVHADNVHDSSHGCCDTKFY 151

tr|Q09JR9|Q09JR9_9ACAR VLFS-DYRSCDVMRGPDG--------NYELWVHSSAINLQSYGCCDTKFA 154

tr|Q09JE7|Q09JE7_9ACAR LLFS-DYRTCDVVLGPSGH-------DYQLWLHAHNVQNGSDPCCDVKFQ 155

tr|Q09JX9|Q09JX9_9ACAR VLYS-DGSTCDVVRLNGQI-------ELWIHSSATSNTGNLNSCCTDKFN 142

tr|Q5GQ85|Q5GQ85_9ACAR VIYT-DGKTCDVVKHGGHT-------ELWVHSSKTS--GGYNNCCDKKFT 140

tr|Q09JS0|Q09JS0_9ACAR VIYS-NYKDCDVMKHTTGD-------EGAVYELWSQNVTKLNPCCSKMFN 165

tr|Q8I9U1|Q8I9U1_9ACAR VVYDVASHHCHVEKLASGA--------YEMWMLEAGGLEVDIECCNKKYD 147

tr|Q8I9U0|Q8I9U0_9ACAR VVYDVPSHHCHIEKLESGA--------YDMWMLEAGGLEVDIECCNKRYD 147

tr|Q5YD59|Q5YD59_ORNMO VVYDSQSHHCHVDKVEKEV-----P-DYEMWMLDAGGLEVEVECCRQKLE 152

sp|Q04669|MOUB_ORNMO LVYDVQSHDCHITKLSSGV--------YQQWQSNGSADDKDIKCCDEKFK 150

tr|Q09JN5|Q09JN5_9ACAR LEFT-NYKDCALFRRPSRQ-----N-GCELWVEKKALQKVPL-CCRFIFD 81

tr|Q09JR1|Q09JR1_9ACAR LAFT-DYETCAIIRVPEKD-------GCQLWTYEEGLRSLNG-YCHFIYN 105

tr|Q8I9U2|Q8I9U2_9ACAR VLYTHLGGGCDVTLFEGQKGQSKVQGPFLELWYHSGASEESMRCCEEEFR 168

tr|Q8I9T9|Q8I9T9_9ACAR LVYS-DDQGCNILQMKTSPFPG----KCELWAPEGKAKNVES-SCSGKFK 158

tr|Q09JJ8|Q09JJ8_9ACAR TVFYADYKTCIVLGLEGVDG------AYELWVKEAKLSGAKA-CCTIVYD 92

tr|Q09JL4|Q09JL4_9ACAR LWMRSGWTTEELDPCCEYI--------YDMWCEGETQKVFDNSTCSMTST 96

: *

tr|Q09JF6|Q09JF6_9ACAR LLCVPVKHYI-----YKRETCLKSRKKSG--------- 124

tr|Q09JV2|Q09JV2_9ACAR LLCGPNKYKV-----YDEEKCKE--------------- 203

tr|Q09JV9|Q09JV9_9ACAR QVTGGNGIR-----DVYQETCPPLPTQ----------- 173

tr|Q09JR9|Q09JR9_9ACAR QVAGGRPIH-----HTWQTYCPPLPRQ----------- 176

tr|Q09JE7|Q09JE7_9ACAR ELVWNRTVF-----QVYHETCPPVPTPVRTL------- 181

tr|Q09JX9|Q09JX9_9ACAR QEKGSRPEH-----VVYRSTCPNLPQ------------ 163

tr|Q5GQ85|Q5GQ85_9ACAR ETRGSTPAN-----EVYK-KCPGMP------------- 159

tr|Q09JS0|Q09JS0_9ACAR ETTQGIASHTVKCPELKKAKTPGRKKKGKKGEVQVEDR 203

tr|Q8I9U1|Q8I9U1_9ACAR ELTSGQVVIR-----PQDKDC----------------- 163

tr|Q8I9U0|Q8I9U0_9ACAR ELTSGQVVIR-----PQDKDC----------------- 163

tr|Q5YD59|Q5YD59_ORNMO ELASGRNQMY-----PHLKDC----------------- 168

sp|Q04669|MOUB_ORNMO ELTSGIDYTK-----PQEKGCETSAK------------ 171

tr|Q09JN5|Q09JN5_9ACAR RLCGSEKHMV-----YNETECTHFYRENRPTLKL---- 110

tr|Q09JR1|Q09JR1_9ACAR VLCGKRKYDV-----YSDQLCGYLDKRREEL------- 131

tr|Q8I9U2|Q8I9U2_9ACAR KNLKEGTAVR-----KVNKNCDYGDVA----------- 190

tr|Q8I9T9|Q8I9T9_9ACAR ELCGDAVETP------YAEGCRVP-------------- 176

tr|Q09JJ8|Q09JJ8_9ACAR ELSKNNKSTV-----ISNYTCSVQQSNAQEAQVLQE-- 123

tr|Q09JL4|Q09JL4_9ACAR KFEKNTTLPWPVLNTPATPAVRAEQKQQKQKP------ 128
